# RETRACTION: Antioxidant and DNA Repair Stimulating Effect of Extracts from Transformed and Normal Roots of *Rhaponticum carthamoides* against Induced Oxidative Stress and DNA Damage in CHO Cells

**DOI:** 10.1155/omcl/9816267

**Published:** 2026-07-30

**Authors:** Oxidative Medicine and Cellular Longevity

RETRACTION: E. Skała, P. Sitarek, M. Różalski, et al., “Antioxidant and DNA Repair Stimulating Effect of Extracts from Transformed and Normal Roots of *Rhaponticum carthamoides* against Induced Oxidative Stress and DNA Damage in CHO Cells,” *Oxidative Medicine and Cellular Longevity*, 2016, 5753139, https://doi.org/10.1155/2016/5753139.

The above article, published online on 29 February 2016 in Wiley Online Library (wileyonlinelibrary.com), has been retracted by between the Journal Editor‐in‐Chief, Jeannette Vasquez Vivar; and John Wiley & Sons Ltd. The retraction has been agreed due to concerns that have been raised with the figures, also reported on PubPeer [1]. Specifically:

• In Figure 6a, the β‐actin/H_2_O_2_ + TR and β‐actin/H_2_O_2_ bands appear unexpectedly similar to the β‐actin/NR and β‐actin/TR bands
• In Figure 6a, SOD2/control band appears highly similar to the CAT/H_2_O_2_ band
• In Figure 6a, SOD2/H_2_O_2_ band appears highly similar to the CAT/H_2_O_2_ + NR band
• In Figure 7a, the SOD2/control band appears highly similar to the CAT/H_2_O_2_ + TR band

The authors responded to the concerns; however, due to the time elapsed since the study, they could not provide the original images for these figures.

After assessing the response to the concerns, we conclude that neither the results nor the conclusions can be considered reliable, and the article is therefore retracted.

The authors disagree with the retraction.
